# 3D Printing of Polycaprolactone–Polyaniline Electroactive Scaffolds for Bone Tissue Engineering

**DOI:** 10.3390/ma13030512

**Published:** 2020-01-22

**Authors:** Arie Wibowo, Cian Vyas, Glen Cooper, Fitriyatul Qulub, Rochim Suratman, Andi Isra Mahyuddin, Tatacipta Dirgantara, Paulo Bartolo

**Affiliations:** 1 Material Science and Engineering Research Group, Faculty of Mechanical and Aerospace Engineering, Institut Teknologi Bandung, Jl. Ganesha 10, Bandung 40132, Indonesia; 2 Research Center for Nanoscience and Nanotechnology, Institut Teknologi Bandung, Jl. Ganesha 10, Bandung 40132, Indonesia; 3 Department of Mechanical, Aerospace, and Civil Engineering, University of Manchester, Manchester M13 9PL, UK; 4 Mechanical Design Research Group, Faculty of Mechanical and Aerospace Engineering, Institut Teknologi Bandung, Jl. Ganesha 10, Bandung 40132, Indonesia; 5 Lightweight Structure Research Group, Faculty of Mechanical and Aerospace Engineering, Institut Teknologi Bandung, Jl. Ganesha 10, Bandung 40132, Indonesia

**Keywords:** 3D printing, electroactive scaffold, polyaniline, tissue engineering

## Abstract

Electrostimulation and electroactive scaffolds can positively influence and guide cellular behaviour and thus has been garnering interest as a key tissue engineering strategy. The development of conducting polymers such as polyaniline enables the fabrication of conductive polymeric composite scaffolds. In this study, we report on the initial development of a polycaprolactone scaffold incorporating different weight loadings of a polyaniline microparticle filler. The scaffolds are fabricated using screw-assisted extrusion-based 3D printing and are characterised for their morphological, mechanical, conductivity, and preliminary biological properties. The conductivity of the polycaprolactone scaffolds increases with the inclusion of polyaniline. The in vitro cytocompatibility of the scaffolds was assessed using human adipose-derived stem cells to determine cell viability and proliferation up to 21 days. A cytotoxicity threshold was reached at 1% wt. polyaniline loading. Scaffolds with 0.1% wt. polyaniline showed suitable compressive strength (6.45 ± 0.16 MPa) and conductivity (2.46 ± 0.65 × 10^−4^ S/cm) for bone tissue engineering applications and demonstrated the highest cell viability at day 1 (88%) with cytocompatibility for up to 21 days in cell culture.

## 1. Introduction

The human body is a complex electrical system that can be most noticeably observed in the action potentials that are integral to the regulatory mechanisms in muscles and nerves. Furthermore, these biological electrical fields—apart from being crucial in the function of tissues such as the heart, muscles, and nerves—have a significant role in cellular behaviour including proliferation, morphology, signalling, migration, orientation, and regenerative processes [1,2,3,4,5,6,7,8]. Subsequently, electrical stimulation techniques and electroactive materials have been developed within tissue engineering in order to modulate cellular responses and enhance tissue regeneration. This new class of smart electroactive biomaterials that are able to deliver electrical, electrochemical or electromechanical stimulation directly to cells, tissue, and organs could bring a significant breakthrough in the tissue engineering field [3,5,6]. Electroactive scaffolds have already been investigated for use in neural [4,5,9], muscle [10,11], bone [12,13,14], and cardiac [15,16] tissue engineering applications. A key requirement is the development of biocompatible, biodegradable, and electrically conductive biomaterials. Electroactive scaffolds can be fabricated from conductive materials or prepared by the addition of conductive fillers to create a composite. These conductive materials can be classified as: carbon-based [5,12,14,15], conductive polymers [1,2,3,6], and metallic-based [16,17,18,19,20,21].

Conductive polymers are electrically active due to a conjugated π-electron backbone allowing for delocalisation and electron mobility. They are attractive due to their facile synthesis routes; excellent electrical and optical properties; flexibility in their electrical, chemical, and physical properties; ability to be functionalised with biological moieties to improve biocompatibility and biodegradability; and wide-ranging processability [3,6,7,8,22,23,24]. A range of conductive polymers have been investigated such as polypyrrole (PPy), polyaniline (PANI), and polythiophene derivatives including poly(3,4-ethylenedioxythiophene) (PEDOT) and poly(3-hexylthiophene) (PHT) [3,6,8,23]. PANI has attracted a considerable amount of attention, especially the half oxidised emeraldine state which is highly conductive and stable, due to the ease of synthesis, low cost production, high stability, and ability to electrically switch from a conductive to a resistive state [3,4]. However, PANI has limitations such as nonbiodegradability and contradictory evidence within the literature regarding the materials biocompatibility. Numerous studies have stated that the material is cytocompatible [25,26,27,28,29,30,31,32] although it may require specific treatments to improve compatibility; however, cytotoxicity and inflammation have also been reported [22,24,33]. Therefore, it is crucial to identify the cytotoxic concentration limit of PANI within a scaffold and procedures to ensure that residual dopants and low molecular weight by-products are not present as these have been suggested to be responsible for the poor biocompatibility [26,33].

Typically, PANI is utilised in a polymeric composite to overcome its inherent brittleness after synthesis. Biodegradable and biocompatible polymers such as polycaprolactone (PCL) [34], poly(ethylene glycol) diacrylate [35], poly(lactic acid) [36], poly(d,l-lactide) [37], gelatin [38,39,40,41], and agarose [27] have been utilised to produce constructs for tissue engineering applications. Further difficulties are observed in processing due to its limited solubility in common organic solvents. PANI based electroactive scaffolds have been fabricated using various conventional fabrication routes such as in situ polymerisation/thermal induced separation [42], solution casting [10,26], hydrogel formation [27,35,39,40,42,43], and electrospinning [11,38,44,45,46]. However, these methods offer limited control of scaffold morphological properties such as pore size, interconnectedness, and fibre diameter. By contrast, 3D printing enables the precise control of scaffold morphology with the production of interconnected structures and a regular pore size [12,13,14]. 3D printing has been utilised in a range of tissue engineering applications such as cartilage [47], bone [48], vasculature [49], and nerve [50] and facilitates the fabrication of multimaterial and geometrical complex architectures that more accurately reflects that native in vivo environment [51].

Herein, we report on the initial development of a novel 3D-printed electroactive scaffold for bone tissue engineering applications. PCL scaffolds incorporating different weight concentrations of PANI was fabricated using a screw-assisted extrusion-based 3D printer. A polymeric composite was chosen to overcome the brittleness and difficulty in processing of PANI with the synthesised PANI particles incorporated through a simple method of melt compounding into the PCL matrix. Key properties of an electroactive scaffold are evaluated including scaffold morphology, conductivity, wettability, and cell viability and proliferation. This initial study focuses on physical, chemical, and electrical characterisation and provides a preliminary biocompatibility assessment for optimisation and development of a 3D-printed electroactive PCL/PANI scaffold.

## 2. Materials and Methods

### 2.1. Materials

Aniline, ammonium peroxidisulphate (APS), hydrochloric acid (HCl), and acetone were purchased from Merck KGaA, Darmstadt, Germany (pro analysis (p.a.) grade). PCL was purchased from Perstorp, Warrington, UK (CAPA 6500, M_w_ 50,000) and is biocompatible and biodegradable polymer with a melting point of 58–60 °C, a glass transition temperature of ~ −60°C, and a density of 1.146 g/mL at 25 °C and was used to prepare composite blends with PANI.

### 2.2. Preparation of Polyaniline

PANI was prepared by a chemical oxidation polymerisation method that has been previously described by Stejskal et al. [52], with slight modification. Briefly, aniline solution (9.1 mL of aniline in 250 mL of 1 M HCl solution) was mixed with APS solution (28.55 g APS in 250 mL of deionised water). After 15 min of stirring, a dark green precipitate was observed that indicated initial formation of PANI in the emeraldine salt state. The mixed solution was left without stirring for 24 h at 15 °C to increase the yield and molecular weight of obtained PANI. The precipitated PANI was collected using a Whatman filter paper (No.42, 2.5 µm pore size) and washed three times with 0.2 M HCl and then acetone to remove initiators, monomers and low-molecular and water-soluble oligomers [53]. Finally, the PANI powder was dried in oven for 24 h at 60 °C.

### 2.3. Polyaniline Characterisation

#### 2.3.1. Fourier-Transform Infrared Spectroscopy

Functional groups of the produced PANI was identified by Fourier-transform infrared spectroscopy (FTIR, IRPrestige-21, Shimadzu, Kyoto, Japan) at room temperature. Samples were measured at wavenumber from 500 to 4500 cm^−1^. Prior to characterisation, a sample pellet was prepared by mixing PANI (10–15 mg) with potassium bromide (KBr powder) (150–250 mg), mortared, and pressed at 700 kN for 5 min.

#### 2.3.2. Powder X-ray Diffraction

The structural phase and crystallinity of the obtained PANI at room temperature was identified by powder X-ray diffraction (XRD, D8 Advance, Bruker, Karlsruhe, Germany) that was performed at room temperature, voltage 40 kV, generator current 35 mA and the scanning scope of angle (2θ) was 5–65°. A CuK radiation with wavelength (λ) of 1.54 Å was used.

#### 2.3.3. Particle Size Characterisation

The PANI powder was wet (dH_2_O) vibratory ball-milled for 5 h to reduce the particle size, increase particle homogeneity, and improve printability. The powder was subsequently lyophilised by snap freezing in liquid nitrogen and dried for 72 h to recover the powder.

The particle size and volume distribution before and after wet ball-milling was quantified using a laser diffraction particle size analyser (Mastersizer 3000, Malvern Panalytical, Malvern, UK). The PANI particles were dispersed in water (refractive index = 1.33). A Fraunhofer scattering and general-purpose analysis model was used. The dispersion was sonicated prior to measurement to minimise bubbles and particle agglomerates. Five measurements were performed. The particle sizes are presented as a volume distribution; Dx(10), Dx(50), and Dx(90), with 10%, 50%, and 90%, respectively, of the particle population lying below these size values.

### 2.4. Fabrication of Polyaniline–Polycaprolactone Scaffolds

The PANI powder (after ball milling) was mixed with the melted PCL (~80 °C) by manual physical melt blending for a minimum of 30 min, based on a previously described method [12,13,14], to ensure a homogenous PCL/PANI blend with PANI filler loading concentrations of 0.1; 1; 2% wt. The PCL/PANI blends were allowed to cool and then cut into small pieces to enable loading into the material chamber of the 3D printer. The composite materials are further mixed and homogenised by the rotation of the screw during extrusion in the screw-assisted extrusion 3D printing process. Mechanical mixing of PANI particles into a PCL solution has been previously described by Wu et al. [54], the process described in this study avoids the use of solvents in the preparation of the composite and is suitable for melt extrusion.

Scaffolds were fabricated using a screw-assisted extrusion-based 3D printing system (3D Discovery, regenHU, Villaz-St-Pierre, Switzerland) with a printing nozzle diameter of 330 µm. The scaffold architecture was designed using computer-aided design software (BioCAD, regenHU, Villaz-St-Pierre, Switzerland). The scaffold was designed with a fibre spacing of 660 µm, a slice thickness of 280 µm, a 0°/90° lay-down pattern, and dimensions of 30 mm × 30 × mm × 3.36 mm. The printing parameters used are deposition velocity of 20 mm s^−1^, a material chamber temperature of 90 °C, an extrusion pressure of 6 bar, and a screw rotation velocity of 15 rpm [12,13,14]. Scaffolds were cut to 11 mm × 11 mm × 3.36 mm for all in vitro studies.

### 2.5. Scaffold Characterisation

#### 2.5.1. Morphology

Scaffold morphology was observed through scanning electron microscopy (SEM, Hitachi S3000N, Hitachi, Tokyo, Japan). Prior to imaging, scaffolds were sputter-coated with platinum for 40 s and imaged using a 10 kV accelerating voltage with both top-down and cross-sectional images of the scaffolds acquired. Images were then analysed using Fiji software, to obtain measurements of pore size and fibre diameter [55].

Scaffold porosity was determined by a gravimetric method [56,57]. Each sample (n = 5) was weighed and the volume based on the printed dimensions before being checked with a calliper. The porosity was determined as follows:

(1) $$P_{scaffold}=\frac{mass}{volume}$$

(2) $$P_{material}=\;X_{PCL}P_{PCL}+X_{PANI}\;P_{PANI}$$

(3) $$Porosity\;\left(\%\right)=\left(1-\;\frac{P_{scaffold}}{P_{material}}\right)\;\times 100$$

where *P_scaffold_* = apparent density of the scaffold, *P_material_* = density of the composite material using the theoretical density of PCL (1.146 g/mL) and PANI (1.33 g/mL [52]), and *X_PCL_* and *X_PANI_* indicate the volume fractions within the composite.

#### 2.5.2. Wettability

The wettability of the PCL/PANI scaffolds was determined through the measurement of the static water contact angle at room temperature using the sessile drop and shape analysis method (KSV CAM 200, KSV Instruments, Helsinki, Finland). A ~13 µL dH_2_O droplet was deposited onto the scaffold (n = 5) and the droplet was imaged by a high-speed camera after 50 s and the contact angle calculated using a Young–Laplace fit.

#### 2.5.3. Mechanical Properties

The mechanical properties of scaffolds were determined at room temperature by compressive testing using a Universal Testing Machine (Tensilon RTF-1310, A&D Company, Tokyo, Japan) at room temperature. Briefly, scaffold samples (n = 3) with dimension ratio of 1:1:1 were prepared and deformed by a crosshead with a load cell of 10 kN and speed of 0.5 mm/min. The stress–strain profiles were obtained from the computational results of the load-displacement calculations. The compressive stress at 10% strain and the compressive Young’s modulus calculated from the gradient of the initial linear elastic region are reported, methodology based on the ISO 844-2014 standard [58].

#### 2.5.4. Conductivity

The electrical conductivity of the PCL/PANI scaffold samples (n = 3) was determined using an Alessi four-point probe at room temperature [11]. A high impedance current source (*I*) was used to supply the current through two outer probes, whilst the voltage (*V*) at the two inner probes was measured with an electrometer. Silver paste was added to improve the contact between probes and scaffolds. The distance between the probes (*L*) is 1 mm and the conductivity (*σ*) of scaffold was determined as follows:

(4) $$\sigma =\;\frac{IL}{VF}$$

where *F* is the correction factors for thickness and geometry of samples.

### 2.6. Biological Evaluation

#### 2.6.1. Cell Culture and Seeding

Human adipose-derived stem cells (hADSCs) (STEMPRO^®^, Invitrogen, Carlsbad, CA, USA) at passage five were used for all in vitro cell culture studies. Cells were cultured in T75 flasks with MesenPRO RS™ media (Invitrogen, Carlsbad, CA, USA), 2% (*v*/*v*) growth supplement, 1% (*v*/*v*) glutamine, and 1% (*v*/*v*) penicillin/streptomycin under standard cell culture incubator conditions (37°C, 5% CO_2_, and 95% humidity). Prior to cell seeding all scaffolds were washed twice in sterile phosphate buffered saline (PBS) before sterilisation by immersion in 80% ethanol for 2 h after which the scaffolds were again washed twice in sterile PBS. The scaffolds were then air dried in a sterile tissue culture laminar flow hood for 12 h. Cells were harvested using trypsinisation (0.25% trypsin/EDTA solution, Sigma-Aldrich, St. Louis, MO, USA) and a cell suspension prepared (0.33 × 10^6^/mL). Each scaffold was then initially seeded with 150 µL of cell suspension (50,000 cells per scaffold) carefully pipetted on top of the scaffold in a non-treated 24-well plate. The scaffolds were then incubated for 2 h under standard cell culture incubator conditions to allow cell attachment before a further 650 µL of supplemented MesenPRO RS™ media was added to obtain a final volume of 800 µL. A non-treated well plate was used to minimise cell migration and attachment to the underlying tissue culture plastic [59]. Cell culture media was changed every three days. All cell-seeded scaffolds were transferred to a new 24-well plate on day 1. Tissue culture plastic (TCP) was used as a control to confirm viable cell culture with the same conditions utilised as the scaffolds

#### 2.6.2. Cell Viability

Viability of the cells cultured on the scaffolds was assessed at day 1 and 21 using a live/dead staining kit (Thermo Fisher Scientific, Waltham, MA, USA), which was prepared according to manufacturer’s instructions. Briefly, a live/dead working solution consisting of a 2 µM calcein acetoxymethyl ester (AM) (live) and 4 µM ethidium homodimer-1 (dead) was prepared in prewarmed PBS. Cell culture media was aspirated, and scaffolds were washed twice in pre-warmed PBS. The live/dead staining solution was added to the scaffold and incubated for 25 min in a cell culture incubator. The scaffolds were then carefully inverted in the well plates using sterile forceps to allow imaging of the top surface of the scaffold. TCP was utilised as a positive control. The scaffolds were imaged using an inverted fluorescence microscope (DMI6000B, Lecia Microsystems, Wetzlar Germany) using two specific filters for green and red fluorescence. Samples (n = 4) were imaged and analysed using Fiji to obtain an average percentage cell viability at day 1 [55].

#### 2.6.3. Cell Proliferation

The proliferation of hADSCs on the scaffolds was assessed using the Alamar Blue assay (also termed the Resazurin assay), which functions through the reduction of resazurin to the highly fluorescent resorufin molecule by metabolically active cells which can be related to cell proliferation. Cell proliferation was assessed on day 1, 3, 7, 14, and 21 for each sample (n = 4) and a TCP control [13]. Briefly, resazurin sodium salt powder (Sigma-Aldrich, St. Louis, Missouri, USA) was used to prepare a stock solution of 0.01% (*w*/*v*) in PBS, which was then filter-sterilised (0.2 µm pore size). At each time point, 80 µL of the stock solution was added directly to each sample well for a final concentration of 0.001% (*w*/*v*). The samples were incubated for 4 h in a cell culture incubator after which 150 µL of each sample was transferred to a 96-well plate. The fluorescence intensity was measured using a microplate reader (Infinite 200, Tecan, Männedorf, Switzerland) at an excitation wavelength of 540 nm and an emission wavelength of 590 nm. Cell culture media containing Alamar Blue was removed and the scaffolds were washed three times in prewarmed PBS before addition of fresh cell culture media and incubation.

### 2.7. Statistical Analysis

Statistical analysis was performed using GraphPad Prism 7.01 statistical software (GraphPad Software, Inc., San Diego, CA, USA). All data is represented as mean ± standard deviation. Comparisons of groups for porosity, wettability, cell viability and cell proliferation data were performed using one-way analysis of variance (ANOVA) and Tukey’s post-hoc test. Significance levels were set at *p* < 0.05 [13].

## 3. Results and Discussions

### 3.1. Characterisation of Polyaniline

**Figure 1:**
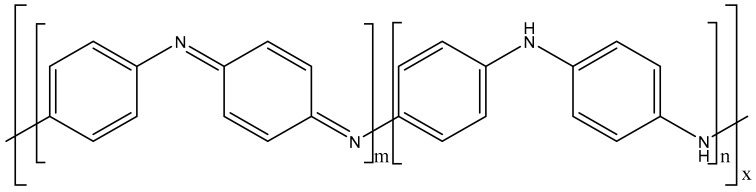
Chemical structure of polyaniline.

The product of the oxidation polymerisation of aniline, PANI (Figure 1), can appear as three idealised oxidation states: leucoemeraldine (fully reduced state, with n = 1 and m = 0), emeraldine (half oxidation state, with n = 0.5 and m = 0.5), and pernigraniline (full oxidation state, with n = 0 and m = 1) [24]. However, only PANI in emeraldine state is able to form a highly electrically conductive PANI upon doping with acid (emeraldine salt) [24]. Therefore, it is necessary to characterise the prepared PANI powder using FTIR and XRD to confirm the successful synthesis of PANI in the conductive form.

**Figure 2:**
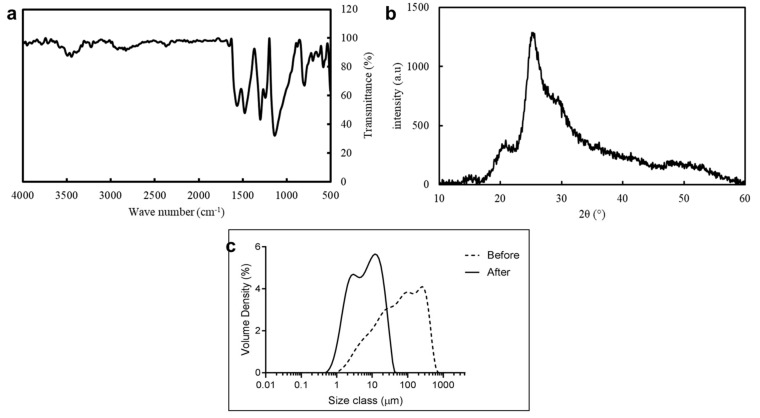
(**a**) Fourier Transform Infra-Red (FTIR) spectra and (**b**) Powder X-Ray Diffraction (PXRD) pattern of prepared polyaniline (PANI) powder, and (**c**) average particle size volume distribution of PANI before and after wet milling for 5 h.

The FTIR spectra of the PANI powder shows several signature peaks of PANI emeraldine salt such as: (i) peaks at 1554 cm^−1^ and 1477 cm^−1^ belonging to the stretching vibration of quinoid (Q) and benzenoid (B) rings of PANI respectively, (ii) peak at 1301 cm^−1^ is correlated with delocalisation of π electrons induced in PANI through protonation, (iii) peak at 1244 cm^−1^ is attributed to C-N stretch vibration in the polaron structure appearing near the secondary amine structure and also as the ribbon characteristic of the protonated form [27] (Figure 2a). Characteristic of the PANI emeraldine salt is a transmittance that continues to rise above the wavelength of 1600 cm^−1^; this is due to the absorption of the free charge carriers in doped polymers [60]. Further characterisation using XRD shows that the sample consists of two broad peaks at 2θ = 15° and 20° representing the amorphous phase and a sharp peak at 25° representing the crystalline phase of PANI (Figure 2b). These peaks are characteristic of PANI in the conductive form (emeraldine salt) and demonstrating the successful synthesis of the polymer [61,62].

The mechanical ball-milling process reduced the PANI particle size and produced a more uniform particle size distribution (Figure 2c and Table 1). Prior to milling the PANI particles size was Dx (90) 306 ± 37 μm, which is too big for printing and can potentially block the nozzle (diameter 330 µm) during extrusion. The particle size was reduced to Dx (90) 20.5 ± 0.1 μm, which is suitable to allow extrusion without blockage. Furthermore, by reducing the particle size the surface area to volume ratio increases which increases the subsequent interfacial interaction and distribution of the PANI filler within the PCL polymer matrix.

### 3.2. Scaffold Characterisation

#### 3.2.1. Scaffold Morphology

**Figure 3:**
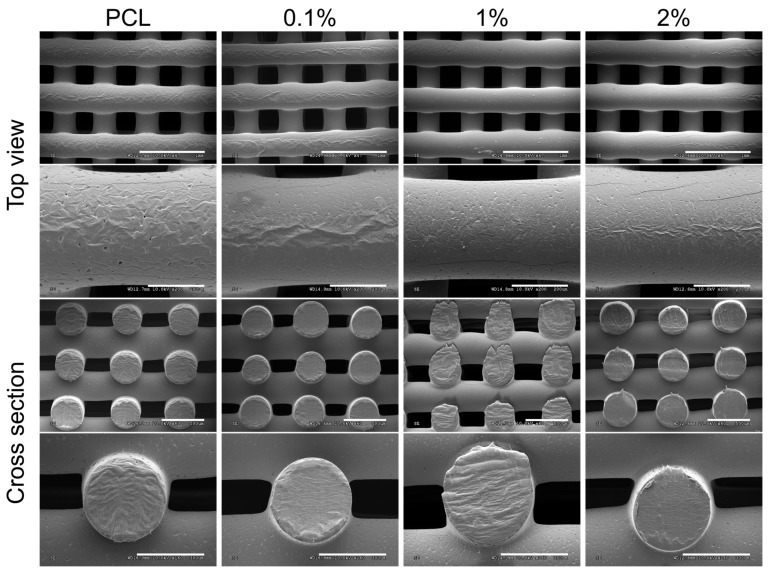
Scanning electron microscope (SEM) images of polycaprolactone (PCL)/polyaniline (PANI) scaffolds with different PANI filler loading (0.1; 1 and 2% wt.). Scale bars from top to bottom: 1 mm, 200 µm, 500 µm, and 300 µm.

Scaffold morphology plays an important role in biocompatibility and cell behaviour. An appropriate morphology is needed to allow the flow and diffusion of nutrients and gases throughout the structure whilst supporting cell attachment, migration, and proliferation during the maturation phase. Preliminary observation using stereomicrograph showed that the scaffolds gradually turned darker with addition of PANI (Figure S1) and the microparticles could be observed within the polymer matrix (Figure S2). The average particle size of PANI observed through the stereomicrographs is 19.9 ± 5.5 μm which is comparable with the particle size of PANI observed after ball-milling (20.5 ± 0.1 μm). This suggests that the PANI particles are well distributed in the PCL matrix and no particle agglomeration occurred during material preparation and 3D printing. FTIR was performed on the PCL/PANI scaffolds and no new peaks were observed confirming that only physical blending occurred, and no new chemical bonds were formed (Figure S3). Further morphological observations of the 3D-printed PCL/PANI scaffolds was performed using SEM and the pore size and fibre diameter quantified (Figure 3 and Table 2). The scaffolds at all concentrations of PANI have a uniform geometry, regular pore size, circular cross-sections of the printed fibres, and are interconnected throughout, which demonstrates successful 3D printing and displays the advantages of 3D printing over conventional fabrication techniques.

The approximate pore size and fibre diameter of the 3D-printed scaffolds are 300 µm and 375 µm, respectively. No significant different in pore size and fibre diameter is observed between the different samples even as the concentration of the PANI filler increases. A pore size of ~300 μm is beneficial for facilitating diffusion of nutrition, allowing cell migration, accelerating cell proliferation, and enabling vascularisation [63,64]. The scaffolds have a porosity of between 44%–50% with a slight decrease in porosity with increasing PANI loading. This is consistent with the larger fibre diameter and smaller pore size observed as PANI loading increases and the higher density of PANI. A high porosity in scaffolds is necessary to allow diffusion and release of biological molecules and nutrients throughout the entire structure that supports appropriate cell behaviour [57,65]. The fabricated PCL/PANI scaffolds have a suitable morphology to enable nutrient diffusion, cell proliferation and migration.

#### 3.2.2. Wettability

**Figure 4:**
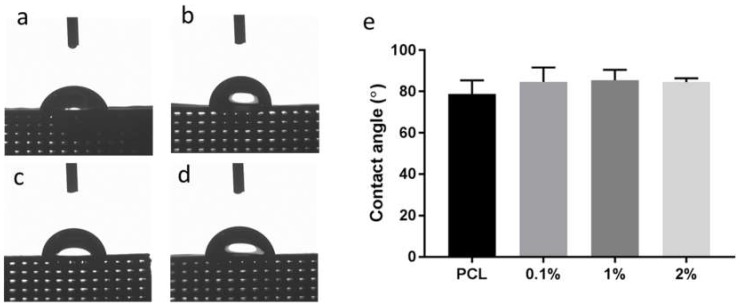
Contact angle images (50 s) of the water droplet on the scaffold with **a**) PCL, **b**) 0.1%, **c**) 1% and **d**) 2% wt. PANI. **e**) Contact angle measurements of the scaffolds.

Scaffold wettability is an important parameter in tissue engineering as it influences the interaction of biological molecules (e.g., protein adsorption) and cells with the biomaterial surface and subsequently has a major role in biocompatibility [66,67,68]. Therefore, the determination of the wettability of the scaffolds was performed by water contact angle measurement (Figure 4).

Material surfaces with a contact angle below 90° are considered hydrophilic, whilst above 90° is considered hydrophobic. Based on the contact angle measurements, a trend is observed with the scaffolds becoming slightly more hydrophobic with increasing concentration of PANI, although not significantly. These results contradict results within the literature that addition of PANI to PCL increases hydrophilicity due to the presence of the -NH_2_ functional group or HCl doping [69,70]. These results could be obtained if PCL and PANI are mixed in a solution phase so that the PANI polymer chain is evenly distributed throughout the matrix and surface. However, when PCL and PANI are mixed in different phases such as in physical melt blending there is the possibility that majority of the water interacts with PCL phase, which is hydrophobic. Thus, the addition of PANI powder into the PCL matrix may not provide a significant hydrophilic effect, which is also observed by Wu et al. who shows that PANI reduces the hydrophobicity of a PCL electrospun mesh but not significantly and has little effect at higher loadings [54]. Although other factors maybe influencing the slight increase in contact angle such as changes in surface roughness of the scaffold. The SEM images of the scaffold surface show a slightly smoother surface with inclusion of PANI with less pits compared to the PCL scaffold (Figure 3). The inclusion of particles effects the material rheology and extrusion flow properties, typically imparting shear thinning behaviour, which can influence the final extruded fibre and surface roughness [71]. Furthermore, the hemispherical and semi-round rod theory of close-packed models predicts that a rougher surface leads to a lower contact angle [72]. Based on this theory, it may be plausible that the addition of PANI results in a smoother surface thus higher hydrophobicity, however, further investigation is required to ascertain this.

#### 3.2.3. Mechanical Properties

**Figure 5:**
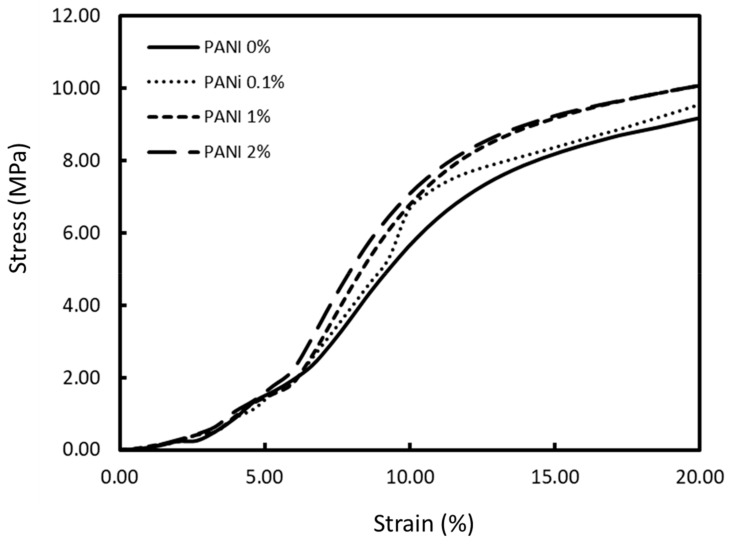
Representative compressive stress–strain curves of the 3D-printed PCL/PANI scaffolds as a function of PANI concentration.

A major drawback of polymeric based scaffolds for load-bearing applications such as bone tissue engineering is their relatively poor mechanical properties. Thus, addition of fillers can be utilised to increase the mechanical properties of the scaffolds. The compressive mechanical properties of the scaffold were determined through uniaxial compression. The scaffolds show mechanical behaviour and stress–strain profiles typical of a cellular solid with an initial linear elastic region, a plastic yielding plateau region, and finally, densification (Figure 5) [73].

The compressive Young’s modulus and strength was calculated based on ISO 844-2014 standard for testing of rigid cellular plastics with the modulus determined as the gradient at the linear elastic region and compressive strength defined at 10% strain (Table 3). The increasing concentration of PANI as filler in the PCL matrix leads to an increase in the compressive strength of the scaffolds. The scaffold with the highest compressive strength was 2% wt. PANI, which is approximately 28% stiffer than PCL alone. The mechanical properties of these scaffolds are within the lower range of human cancellous bone, which has wide range of compressive strength (1–12 MPa) and Young’s modulus (100–5000 MPa), dependent on anatomical location, age, and measurement technique [74,75]. The scaffolds may have mechanical suitability for specific cancellous bone applications.

#### 3.2.4. Conductivity

Scaffold conductivity is a major parameter for a successful electroactive scaffold. A low conductivity biodegradable polymer, such as PCL, requires the addition of a conductive filler (PANI in this study) to sufficiently increase the scaffold conductivity so it may be utilised for electrical stimulation in tissue engineering applications. The conductivity of the scaffolds was measured using the four-point probe method (Table 4). The conductivity of PCL was unquantifiable; Chen et al. also used a four-point probe, and likewise showed undetectable conductivity [11]. However, PCL has been shown to be approximately 1.1 × 10^−11^ S/cm at 20 ± 2 °C [54]. The inclusion of 0.1% wt. PANI significantly increased the conductivity (2.46 ± 0.85 × 10^−4^ S/cm) of the scaffold compared to PCL alone. Increasing the PANI filler concentration further only gave a small increase in conductivity. This may be due to the critical filler concentration—the percolation threshold—being reached and a continuous electrical network formed which overcomes the insulating properties of the PCL matrix. Further investigation at lower and higher concentrations is required to determine the specific percolation threshold within this specific composite. The conductivity of PCL/PANI scaffolds is within the region of conductivity observed in cancellous (1.6–2.0 × 10^−3^ S/cm) and cortical bone (5.8–6.3 × 10^−4^ S/cm) [76]. The electrical conductivity could be further improved by using a solvent mixing method to improve the particle distribution or increasing the aspect ratio of the PANI to form fibres, thus improving the electrical network [34]. Furthermore, the long-term conductivity profile of the scaffolds in aqueous physiological conditions still needs to be assessed to determine the rate of de-doping of the PANI and the subsequent decrease in conductivity.

The addition of PANI improves the conductivity of the printed scaffold and is potentially suitable for applying electrical stimulation to guide cell behaviour. By improving the conductivity of the scaffold, the applied voltage or current could be reduced, but could still achieve the desired effect in the target cell or tissue.

### 3.3. Cell Viability and Proliferation

The 3D-printed PCL/PANI scaffolds have been initially biologically evaluated by assessing the cell viability and proliferation of seeded hADSCs and to determine the maximum PANI particle loading cytotoxicity threshold. As previously discussed, the cytocompatibility of PANI has been shown to be mostly positive but studies have demonstrated significant cytotoxicity. This contradiction needs further evaluation and the determination of a cytotoxicity threshold or if other factors are implicated, such as toxic by-products.

**Figure 6:**
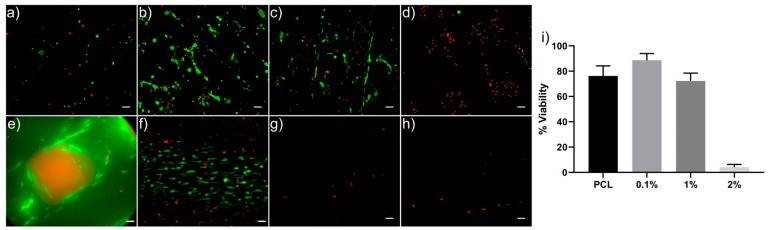
Cell viability of the PCL/PANI scaffolds. Live (green) and dead (red) staining of the hADSCs after 1 day of cell culture (**a**) PCL, (**b**) 0.1%, (**c**) 1%, and (**d**) 2% wt. PANI (scale bar = 100 µm); and 21 days (**e**) PCL, (**f**) 0.1%, (**g**) 1%, and (**h**) 2% wt. PANI (scale bar = 50 µm). (**i**) Percentage cell viability after 1 day in cell culture.

The cell viability of the PCL/PANI scaffolds was assessed using a Live/Dead assay after 1 and 21 days in cell culture (Figure 6). The results demonstrate that at day 1, the PCL, 0.1% and 1% wt. PANI scaffolds have high cell viability with only a few dead cells observed and percentage viability of approximately 75%, 88%, and 75%, respectively. The 2% wt. PANI scaffold showed a clear cytotoxic effect with only a few live cells present. Higher concentrations of PANI seem to generate a cytotoxic response in hADSCs. Noticeably, the cell morphology on the 0.1 and 1% wt. PANI scaffolds are more spread out and have larger cell bodies compared to pure PCL, which may be a result of changes in the surface chemistry and roughness of the scaffolds. This warrants further investigation to understand the biomaterial–cell interactions influencing cell adhesion and spreading. Cell viability by day 21 demonstrates that only the PCL and 0.1% wt. scaffolds support viable cells. The scaffolds show only a few dead cells most likely due to the scaffolds supporting a large amount of cell proliferation and the cells reaching confluency. The 1% and 2% wt. scaffolds show no viable cells.

**Figure 7:**
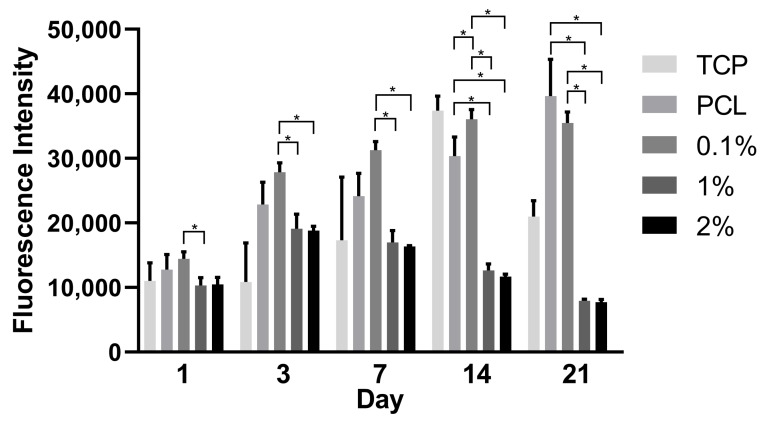
Proliferation of human adipose derived stem cells (hADSCs) on the TCP and PCL/PANI scaffolds measured using the Alamar Blue assay at day 1, 3, 7, 14, and 21. Results indicate significant difference in proliferation between samples (* *p* < 0.05).

Cell proliferation of hADSCs on the PCL/PANI scaffolds was evaluated by the Alamar Blue assay up to 21 days (Figure 7). All scaffolds show successful cell attachment on day 1 with comparable fluorescence intensities to the TCP control. The PCL and 0.1% wt. PANI scaffolds supported cell proliferation for up to 14 days with the 0.1% wt. PANI scaffold have the highest cell proliferation by day 14. This suggests that the presence of PANI at low percentage weight-loading increases cell proliferation. At day 21, a slight decrease in fluorescence intensity is observed in the 0.1% wt. PANI scaffold whilst proliferation increases in PCL only. However, a similar decrease in fluorescence intensity was also observed on the TCP. This phenomenon is most likely due to cell confluence being reached after 21 days of cell culture and the metabolic activity of the cells decreasing thus a decrease in fluorescence intensity, as indicated by the Alamar Blue assay. Furthermore, this indicates that a confluent cell state was potentially reached earlier in the 0.1% wt. PANI scaffold than the PCL only scaffold.

However, both 1 and 2% wt. PANI scaffolds showed only an increase in fluorescence intensity until day 3 before decreasing, indicating that the scaffolds at these levels of PANI loading are cytotoxic. This cytotoxicity is seen immediately at the higher concentration as indicated by the cell viability imaging but is delayed in the 1% wt. scaffold, which at day 1 has high cell viability (Figure 6). The cytotoxicity may be caused by the release of dopant residues or low molecular weight by-products generated during PANI synthesis and at higher PANI concentrations is mostly toxic with 24 h, but at lower concentrations requires time to leach out into the cell culture media before reaching their toxic level and causing a cytotoxic effect. Similar findings have shown that the reaction residues and by-products are a likely cause for cytotoxicity [26,32]. Furthermore, PANI dopants have been demonstrated to leach out at physiological pH [77]. Changes in pH, weight loss, and chloride ion concentration in the PCL/PANI scaffolds during incubation in PBS for up to 14 days are demonstrated in Table S1, Table S2 and Figure S4, respectively. Small changes in the pH of PBS solution and the weight of the scaffold are observed after 14 days. The addition of PANI does not significantly alter the rate of degradation between samples within the timeframe of this study. The chloride ion concentration in the 0.1% wt. PANI scaffold and the 1% wt. PANI scaffold at day 1 and day 14 are relatively similar. However, the chloride ion concentration significantly increases in the 2% wt. PANI scaffold which may have a role in the cytotoxicity observed in these samples. Nonetheless, the trend of pH and chloride ion concentration are not consistent with cell proliferation results, suggesting that pH or leaching of chloride ion is not the main cause of decreasing cytocompatibility as PANI concentration increases. Humpolicek et. al. found that biocompatibility of PANI can be increased by repeated deprotonation and reprotonation cycles, indicating that the release of by-products of the polymer synthesis is the main cause of cytotoxicity of PANI rather than PANI itself [32]. This study assessed cell proliferation up to 21 days, which is longer than other studies [26,32,54,78] and may allow the build-up of cytotoxic effects that might not be observed during shorter studies. Therefore, further investigation is required to ascertain the role of dopant or by-product release on biocompatibility and additional purification and washing steps of the samples may be required. Furthermore, secondary biological effects of PANI as a nonbiodegradable material, to the authors knowledge, is not known and should be investigated in further studies before PCL/PANI scaffolds can be determined as fully viable.

## 4. Conclusions

This study demonstrates—for the first time—the fabrication of a 3D-printed electroactive PCL/PANI composite scaffold and its initial development and characterisation for tissue engineering applications. The synthesis of the conductive emeraldine PANI was successful and the powder was ball milled to a suitable size for extrusion-based 3D printing and melt-blending with PCL. The scaffold wettability and mechanical properties are similar to PCL alone, however, the electrical conductivity is significantly higher, demonstrating its suitability as an electroactive scaffold. A preliminary biological assessment of scaffold cytocompatibility was conducted with hADSCs, which showed a cytotoxic effect at higher concentrations (1 and 2% wt.) of PANI after 3 days of cell culture, while the 0.1% wt. PANI scaffolds were cytocompatible and had comparable cell proliferation to PCL-only scaffolds for up to 21 days. Scaffolds with 0.1% wt. PANI show cytocompatibility and are conductive. Further investigation is required to elucidate the mechanism of cytotoxicity and improve the conductivity of the scaffolds. However, this is a promising step in the initial development and optimisation of a 3D-printed electroactive polymeric composite scaffold for bone tissue engineering applications and allows further investigation within the field.

## Figures and Tables

**Table 1:** Particle size of polyaniline before and after wet ball-milling for 5 h.

Volume Distribution	Size (µm)	
	**Before**	**After**
Dx (10)	6.57 ± 0.142	1.66 ± 0.004
Dx (50)	62.4 ± 3.3	6.47 ± 0.06
Dx (90)	306 ± 37	20.5 ± 0.1

**Table 2:** Summary of average fibre diameter and pore size of PCL/PANI scaffolds based on SEM and porosity determined gravimetrically (n = 5).

PANI Concentration (% wt.)	Fibre Diameter (µm)	Pore Size (µm)	Porosity (%)
0	379.1 ± 24.1	314.6 ± 23.8	49.51 ± 0.740
0.1	364.8 ± 22.9	305.9 ± 35.5	48.16 ± 1.071
1	379.3 ± 21.2	295.5 ± 34.5	48.01 ± 0.457
2	382.5 ± 22.2	290.9 ± 28.6	44.69 ± 1.602

**Table 3:** The compressive Young’s modulus and compressive strength at 10% strain of the PCL/PANI scaffolds.

PANI Concentration (% wt.)	Compressive Young’s Modulus (MPa)	Compressive Strength (MPa)
0	64.43 ± 3.97	5.53 ± 0.08
0.1	68.35 ± 5.15	6.45 ± 0.16
1	73.83 ± 4.22	6.83 ± 0.10
2	82.61 ± 6.94	7.38 ± 0.35

**Table 4:** Conductivity of the PCL/PANI scaffolds.

PANI Concentration (% wt.)	Conductivity (10^−4^ S/cm)
0.1	2.46 ± 0.85
1	2.53 ± 0.65
2	2.84 ± 0.51

## Supplementary Materials

The following are available online at https://www.mdpi.com/1996-1944/13/3/512/s1, Figure S1. Optical microscopy images of the 3D-printed PCL/PANI scaffolds: a) PCL, b) 0.1%, c) 1.0% and d) 2.0% wt. PANI (scale bar = 1 mm); Figure S2. Optical microscopy of scaffold with 0 and 0.1 wt.% PANI scaffold highlighting the observable inclusion of the PANI microparticles within the PCL matrix (Scale bar = 0.2 mm); Figure S3. FTIR spectra of PANI, PCL and PANI/PCL scaffold. Suggesting that no new bonds are formed between PCL-PANI in the composite scaffold, this implies that only physical blending of PANI and PCL occurred in the PCL/PANI scaffold; Figure S4. Determination of chloride ion concentration in phosphate buffered saline (PBS) solution after immersion of PCL/PANI scaffolds for 1 and 14 days; Table S1. pH observation of phosphate buffered saline solution after immersion of scaffolds as a function of immersion time; Table S2. Scaffold weight loss during incubation in PBS solution for up to 15 days.
