# Supplementary material for: 3D Printing of Polycaprolactone–Polyaniline Electroactive Scaffolds for Bone Tissue Engineering
Source: Materials (Basel). 2020 Jan 22;13(3):512. doi: 10.3390/ma13030512 (PMC7040705; doi:10.3390/ma13030512)
Supplement: Supplementary file 1 [file materials-13-00512-s001.pdf]

materials

Supplementary Materials

3D Printing of Polycaprolactone–Polyaniline Electroactive Scaffolds for Bone Tissue Engineering

Arie Wibowo [1,2,*,†], Cian Vyas [3,*,†], Glen Cooper [3,*], Fitriyatul Qulub [1], Rochim Suratman [1], Andi Isra Mahyuddin [4], Tatacipta Dirgantara [5] and Paulo Bartolo [3]

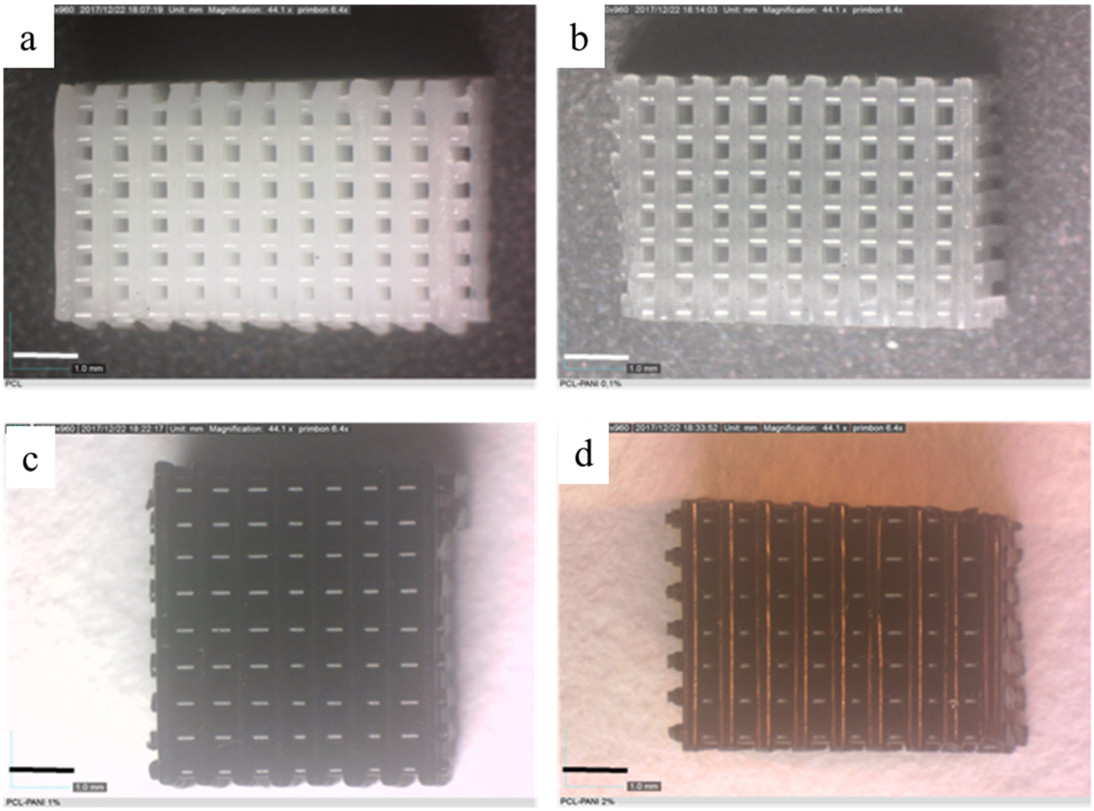

Figure S1. Optical microscopy images of the 3D printed PCL/PANI scaffolds: **a**) PCL, **b**) 0.1%, **c**) 1.0% and **d**) 2.0% wt. PANI (scale bar = 1 mm).

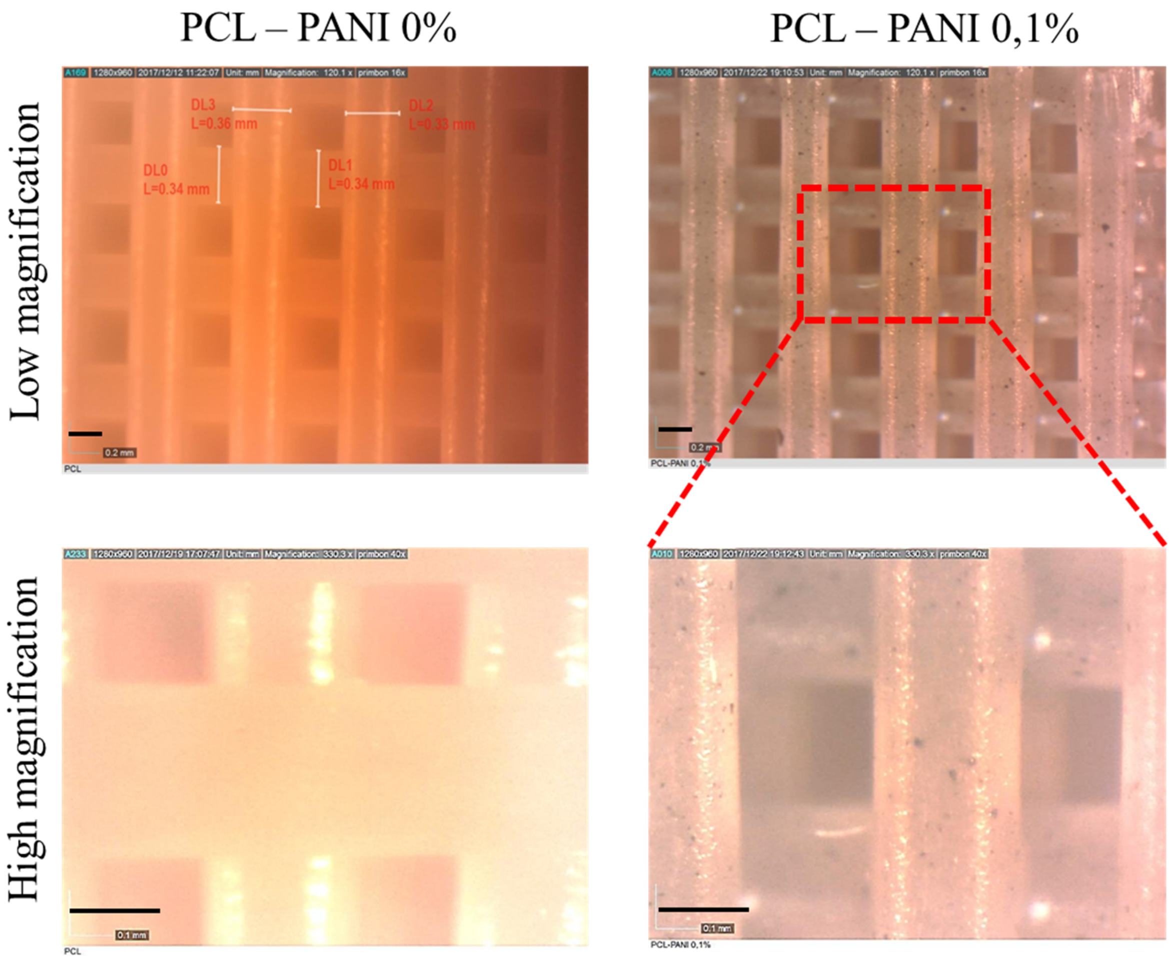

Figure S2. Optical microscopy of scaffold with 0 and 0.1 wt.% PANI scaffold highlighting the observable inclusion of the PANI microparticles within the PCL matrix. Scale bar = 0.2 mm.

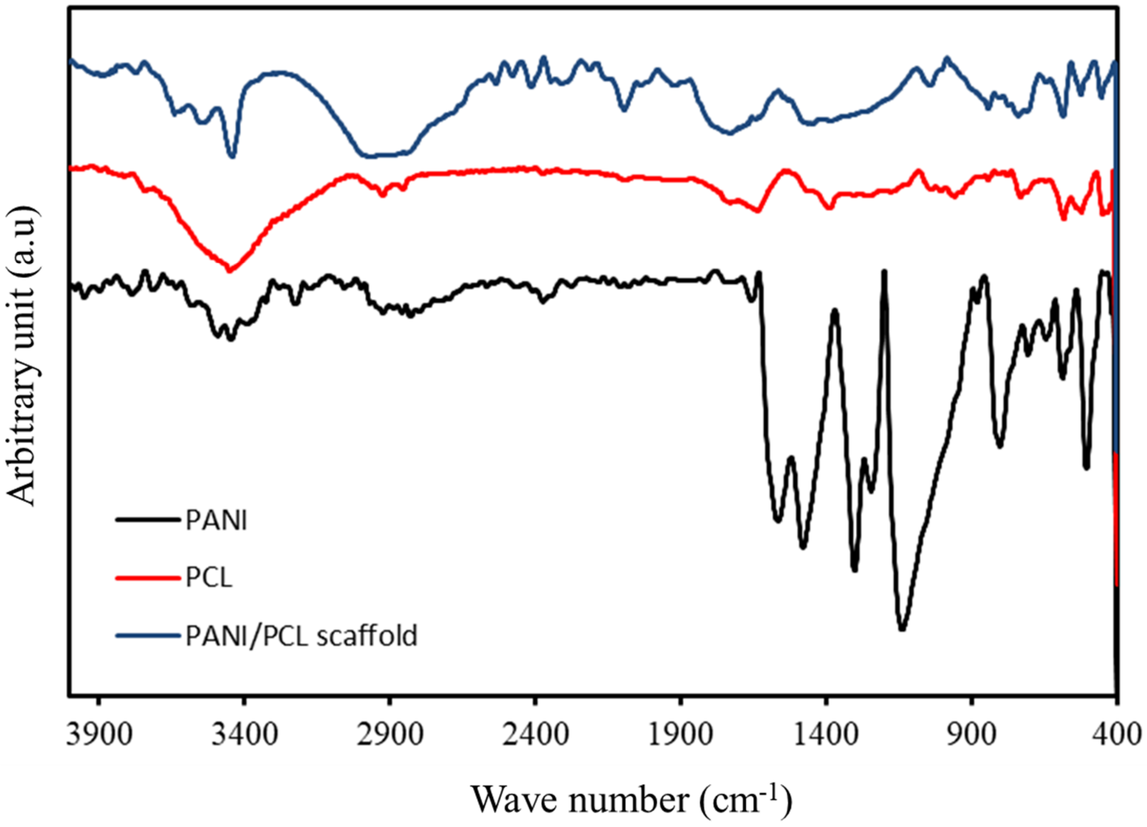

Figure S3. FTIR spectra of PANI, PCL and PANI/PCL scaffold. Suggesting that no new bonds are formed between PCL-PANI in the composite scaffold; this implies that only physical blending of PANI and PCL occurred in the PCL/PANI scaffold.

Table S1. pH observation of phosphate buffered saline solution after immersion of scaffolds as a function of immersion time (n = 3).

Incubation time (days)	Samples			
	**PCL-PANI 0%**	**PCL-PANI 0.1%**	**PCL-PANI 1.0%**	**PCL-PANI 2.0%**
0	7.45 ± 0.02	7.45 ± 0.01	7.45 ± 0.01	7.45 ± 0.01
1	7.45 ± 0.01	7.45 ± 0.02	7.45 ± 0.02	7.45 ± 0.01
3	7.44 ± 0.01	7.44 ± 0.01	7.44 ± 0.01	7.44 ± 0.02
5	7.44 ± 0.01	7.44 ± 0.01	7.44 ± 0.01	7.44 ± 0.01
7	7.34 ± 0.01	7.35 ± 0.01	7.34 ± 0.01	7.33 ± 0.01
9	7.34 ± 0.01	7.36 ± 0.01	7.31± 0.02	7.32 ± 0.02
11	7.33 ± 0.02	7.34 ± 0.01	7.31 ± 0.01	7.30 ± 0.01
14	7.31 ± 0.01	7.33 ± 0.03	7.28 ± 0.01	7.27 ± 0.01

Table S2. Scaffold weight loss during incubation in PBS solution for up to 15 days (n = 3).

Sample	Remaining mass of scaffold (%) as incubation time (days)					
	**0**	**3**	**6**	**9**	**12**	**15**

PCL-PANI 0.0%	100.00 ± 0.23	99.92 ± 0.08	99.84 ± 0.16	99.23 ± 0.19	99.23 ± 0.25	98.96 ± 0.10
PCL-PANI 0.1%	100.00 ± 0.13	99.56 ± 0.18	99.47 ± 0.24	99.29 ± 0.32	99.35 ± 0.22	98.99 ± 0.22
PCL-PANI 1.0%	100.00 ± 0.24	99.66 ± 0.29	99.70 ± 0.35	99.16 ± 0.17	98.65 ± 0.34	98.82 ± 0.29
PCL-PANI 2.0%	100.00 ± 0.25	99.68 ± 0.22	99.78 ± 0.20	99.39 ± 0.23	99.39 ± 0.23	98.80 ± 0.17

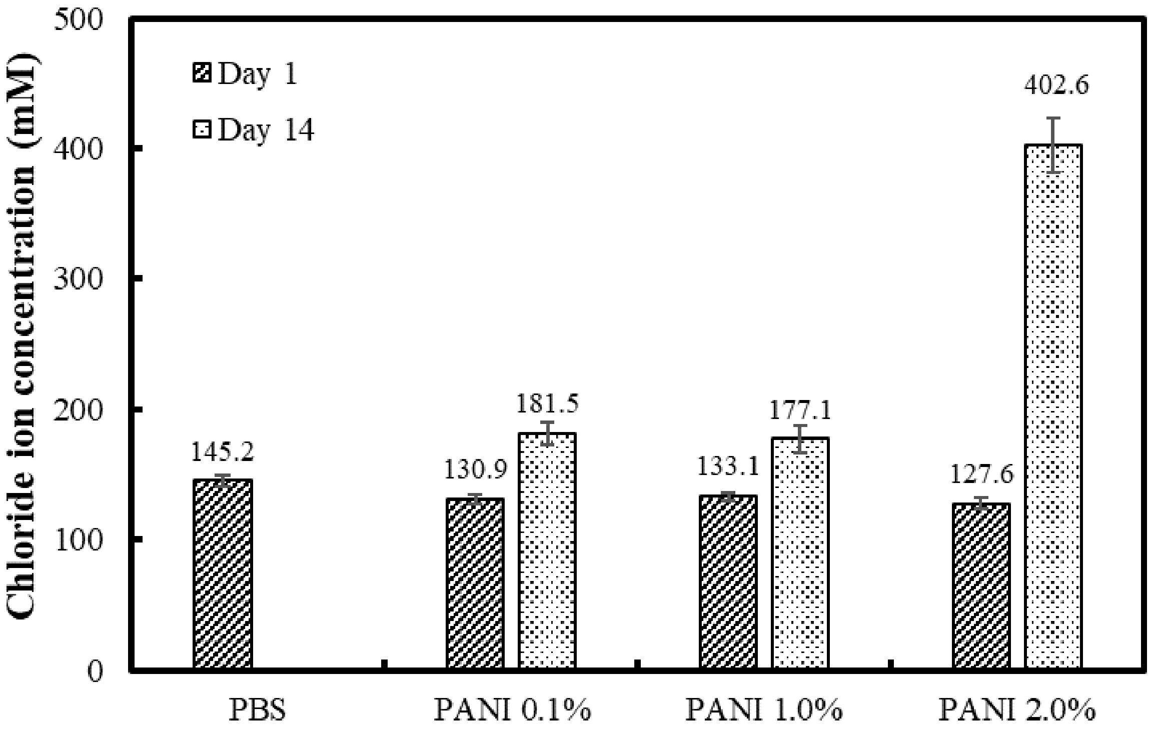

Figure S4. Determination of chloride ion in phosphate buffered saline (PBS) solution after immersion of scaffolds for 1 and 14 days (n = 3).

© 2020 by the authors. Licensee MDPI, Basel, Switzerland. This article is an open access article distributed under the terms and conditions of the Creative Commons Attribution (CC BY) license (http://creativecommons.org/licenses/by/4.0/).
